# High burden of atopy in immigrant families in substandard apartments in Sweden – on the contribution of bad housing to poor health in vulnerable populations

**DOI:** 10.1186/s40413-018-0188-1

**Published:** 2018-05-15

**Authors:** Jens Christian Richter, Kristina Jakobsson, Tahir Taj, Anna Oudin

**Affiliations:** 1 Division of Occupational and Environmental Medicine, Medical Faculty, Lund University, 22185 Lund, Sweden; 2 Department of Respiratory Medicine and Allergology, Lund University Hospital, Region Skåne, Lund, Sweden; 3 Department of Occupational and Environmental Medicine, Göteborgs Universitet, Gothenburg, Sweden

## Abstract

**Background:**

Atopic disorders are a global concern. Studies in migrant populations can illuminate the interplay of genetic and environmental factors. Exposures related to bad housing (indoor dampness, mould growth, crowding etc.) are likely to play a role in how socioeconomic inequalities can turn into health disparities for disadvantaged populations. The sizable immigrant population living in very poor-quality housing in Malmö, Sweden, became the focus of a cross-sectional study.

**Objective:**

To describe atopic disorders and sensitizations in a population living in substandard housing in Malmö, Sweden, with an emphasis on their relation to harmful exposures from the built environment.

**Methods:**

Families were recruited via identification of any children with symptomatic airway afflictions from health care records, and also asymptomatic children from school lists. Interviewer-led health questionnaire data and data from self-reports about living conditions were obtained together with data from home inspections carried out by health communicators. Families underwent skin prick tests (SPT) against common aeroallergens.

**Results:**

As could be expected from background demographic information, it turned out that we effectively studied an immigrant population inhabiting very precarious housing outside the center of Malmö. A total of 359 children from 130 families (total 650 participants) were included. Overall the prevalence of potentially harmful environmental exposures was high (signs of moisture or mould in more than 50% of apartments, indoor smoking in 37% of households). Atopic disorders were common among both adults and children. SPTs showed a spectrum of sensitizations consistent with unselected populations in Sweden. Paternal sensitization in the SPT was associated with higher risk of sensitization for offspring than maternal sensitization. Few statistically significant associations of atopic sensitization with studied environmental exposures were detected (for example objective signs of dampness /mould in bathrooms). There were marked discrepancies between asthma diagnoses obtained from the health records and parental reports of such diagnoses and treatment for their children.

**Conclusions:**

The atopic burden in this selected immigrant population was high, and results point to unmet medical needs. Health care systems caring for such populations need to be aware of their specific health needs; comprehensive asthma and allergy care should include consideration of harmful environmental exposures, adhering to the precautionary principle.

## Background

Allergic diseases and sensitizations are issues of global importance for clinicians. Atopic disorders are common and international studies such as ISAAC (The International Study of Asthma and Allergies in Childhood) [1, 2] have shown that their prevalence differs between countries and that their distribution within countries can differ widely, as well. Studies in migrant populations have the potential to shed light on the complex interactions between genetic and environmental factors that are at play in shaping the atopic phenotype [3]. The temporary or permanent transplantation of individuals from one country into another can be interpreted as a natural immunological experiment. It has been noted that internationally adopted children and children of immigrants have higher rates of asthma and other atopic disorders than would be expected in comparable children in their country of origin [4]. Numerous other factors are expected to exert an influence on children’s risk for atopic disorders, such as duration of breastfeeding [5, 6]; parental smoking, especially maternal smoking during pregnancy; sibship size and birth order [7, 8]; parental atopy; daycare attendance [9, 10]; degree of acculturation and adoption of lifestyle changes [11, 12].

Also of relevance are factors related to the built environment (dampness and mould in dwellings) [13–15], and air pollution [16]. Many of these factors obviously co-vary with socioeconomic status and broader neighbourhood variables [17, 18].

As the drivers for international migration, such as global environmental change, conflict, and economic inequalities are likely to become stronger in the future, understanding and supporting the health of immigrants remains among the more challenging tasks of public health practitioners. Studies on health and international migration are of great interest for policy makers, health system planners, and clinicians. Such enquiries are also relevant to social scientists, environmental and urban planners, housing administrators, and the economic sector.

In general terms, there have been observations that the health of immigrants in the first generation is better than that of a comparable population in the country of arrival (this has been termed the “immigrant paradox”) [19, 20]. The advantage however is lost more or less quickly, especially as lifestyles of high-income countries are adopted and presumably as environmental exposures act on the new arrivals [19, 20]. Poverty and economic disadvantages are common in immigrant populations, which can lead to adverse health outcomes along numerous pathways, such as housing/residential areas with harmful environmental exposures, and limited access to quality healthcare. The importance of the built environment for health has been recognized for centuries, and in recent decades much attention has been focused on indoor dampness and mould exposures. There is broad consensus that dampness and mould in apartments have negative effects on the health of the inhabitants [18], especially in regard to respiratory diseases, and here especially for children [21]. It is biologically plausible that exposure to indoor allergens plays a role in atopic sensitization and in clinical outcomes such as symptomatic asthma. Several intervention studies have addressed this problem. If interventions are to work at all, they must combine allergen avoidance/minimization measures with education, lifestyle interventions, and conclusions from these studies indicated that as such, multi-trigger, polysectoral interventions are best delivered through community health workers [22–24].

### Description of background and study area in Malmö, Sweden

**Fig. 1:**
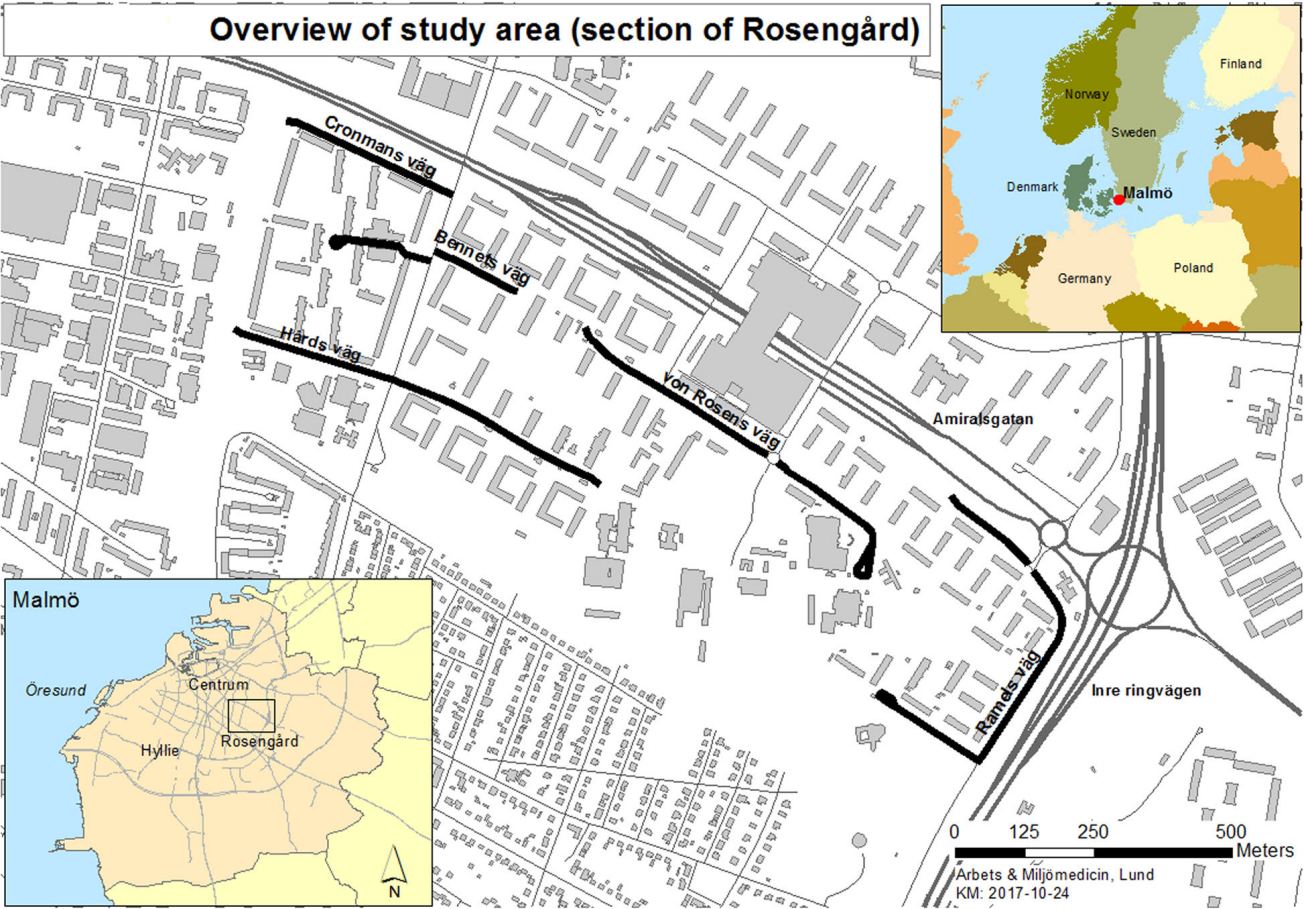
Overview of study area (the involved streets are marked in black), and surrounding urban layout, plus context zoom in Northern Europe

In Sweden, the number of immigrants has been high for many decades (www.migrationsverket.se), and there are a number of differences between the immigrant populations and the resident Swedish population in terms of poverty, health outcomes and educational outcomes. Both in the larger cities and in smaller towns, there are some predominantly immigrant neighbourhoods, that are characterized by social (and ethnic) segregation, The city district of Rosengård in Malmö (see Fig. 1) is relatively close to the city centre, with an official number of 22,000 inhabitants in 7600 households (all population data retrieved from www.malmo.se). It is separated into several distinct parts and contains mainly apartment blocks with 3 to 9 floors; population statistics for Malmö show that the proportion of first and second generation immigrants in Rosengård is high at 86% of the total population compared to 37% for the whole of Malmö; Iraq, former Yugoslavia, Albania, Syria, Lebanon, Afghanistan, and Somalia are the dominant countries of origin. The proportion of children who live in poverty is 62%, and 96.5% of all children have a foreign background (themselves born abroad or at least one parent born abroad), [25]. Within Rosengård, there are marked differences in the quality and residential desirability of neighbourhoods and buildings. Particularly, in one part, called Herrgården (initially planned for 3000 inhabitants, official population 5000, but estimated at closer to 8000), a large number of apartment buildings in a single street had changed private ownership several times after the deregulation of the Swedish housing market in the 1980’s. These owners seriously neglected upkeep and renovation of these buildings [26]. The quality of the buildings deteriorated over many years, and in 2008 the attention of the public was focused by an investigative television programme depicting overcrowded, damp apartments covered in mould, infested by cockroaches and other vermin, non-functioning ventilation, and sanitary facilities, etc. Over the following months, inspections by building engineers confirmed widespread structural and functional deficiencies. At the end of the litigation that followed, the owner was ordered to renovate all affected apartments (*n* = 867) in the area.

The present study is part of an underlying and ongoing study conducted since 2009 by the Division of Occupational and Environmental Medicine of Lund University, Sweden, further described elsewhere. [27, 28] (http://dx.doi.org/10.5061/dryad.802hc/1). The study was developed and implemented with a twofold aim: 1) describe the health (in its social context) of a disadvantaged population in Sweden and 2) research the effects which renovations in apartments with multiple structural problems (“bad housing”) would have on respiratory and other health outcomes of children and families living in the described apartments in Rosengård.

The aim of the part of the study presented here was to describe atopic disorders and sensitizations in the factually immigrant population living in Herrgården and in the adjoining area with a focus on their relationship with exposure to bad housing.

## Methods

### Study area

The context of the study area was described above.

The study area was defined as the described area in Rosengård, namely the street in Herrgården where all affected apartments were located (see Fig. 1), as well as several adjoining streets with similar infrastructure and sociodemographic composition.

### Participants

The recruitment and baseline characteristics of the participants have been further described elsewhere [27, 28] (http://dx.doi.org/10.5061/dryad.802hc/1). In short, electronic patient registries at local health centers were searched for all children between the ages of 0 and 13 years with diagnoses of asthma or other respiratory diseases, that were registered as living in the defined study area at the time of the documented healthcare contact.

In order to expand recruitment beyond an enriched sample of children with respiratory health issues, the sampling frame was redesigned to include children from the study area who were aged between 6 and 13 years from class lists (class grades 0 to 5) which were obtained from the local school, i.e., non-selected with regard to health outcomes.

All families of the children received written invitations from the researchers to participate in the study (for details of recruitment see [28]. It was explained that the study would entail a home visit, and that data would be collected on the children and also other family members living in the same household.

The primary aim of the study was to describe a population living in precarious housing conditions. It was known that the population in the study area consisted almost exclusively of immigrant families. This was, however not an inclusion criterion.

Data for participants from both the area with structurally deficient housing (Herrgården) and the area with intact housing (Törnrosen) were analysed together because we aimed to describe the population as a whole, in terms of overall burden of exposures and atopic outcomes.

### Data collection – Home visits

If families chose to participate, a home visit was scheduled during which pairs of health communicators fluent in the family’s native language visited the apartment.

At the start of the home visit, informed written consent (in the native language of the family) for participation in the study was obtained from the parents. Ethical approval for the study was granted by the Regional Ethical Review Board through Lund University (Registration number 2010/212).

### Questionnaires and study instruments

Interviewer-led questionnaires were used to collect sociodemographic information on all core family members in the household, physical apartment characteristics (number of rooms, number of regular occupants, presence of pests), subjective exposure assessment in the various rooms (dampness, mould), information about lifestyles and indoor air pollution (e.g. smoking habits), allergic/atopic diseases of the parents,. In addition, questionnaires were used to collect health information for all children aged 0-13 years, with the main focus on respiratory and allergic symptoms. The mothers were also asked about breastfeeding practices for all children. Lastly, a standardized visual assessment of multiple areas of all homes was carried out at the time of the home visit by the health communicators, who had undergone training in home assessment. Documented in the form of a checklist, the assessment scored the extent of visible moisture damage, visible mould, and mould odour in all rooms.

### Skin prick tests

The skin prick tests (SPT) were performed by experienced allergy nurses at the local community centre after the home visit. Families were asked to come to the SPT sessions with as many family members as possible. Skin prick tests with a standard panel for aeroallergens (ALK-Abelló S.A., Madrid), including moulds, house dust mites, plants, animal dander, and, in addition, cockroach (Bla g 2), were performed on all participants that were willing to be included. All tests were performed following usual standards and procedures.

### Statistical analysis

Descriptive data analysis includes frequencies for categorical data and means, medians, ranges, and standard deviations for numerical data. Categorical data was analysed with chi-squared tests for comparisons. Where appropriate, numerical data were compared between groups with independent t-tests or one-way ANOVA. A *p*-value of less than 0.05 was considered significant. All statistical analyses were performed with IBM SPSS Statistics Version 20.0 (IBM Corporation, Armonk (NY), USA). Raw annotated data has been made publicly available in conjunction with a prior publication [28] (http://dx.doi.org/10.5061/dryad.802hc/1).

## Results

The families of the participating children yielded an overall number of 650 participants from 130 families (core family members), which were included in the study (shown in Table 1).

**Table 1:** Demographic data and selected health-related information on study participants

	All Children	Male children	Female children	Mothers	Fathers
N (650)	359	190 (53%)	169 (47%)	130	100
Born in Sweden	318 (89%)	169 (89%)	150 (89%)	0	0
Age(yrs ± SD)	7.06 (3.8)	6.9 (3.8)	7.3 (3.8)	32.7 (6.1)	39.3 (9.0)
Crowded living quarters (%)	255 (71%)	133 (70)	122 (72)	90 (69)	73 (73)
Asthma (phys-diag.)	83 (23%)	50 (26%)	33 (19.5%)	6 (5%)	7 (7%)
Asthma medication	54 (15%)	35 (18%)	19 (11%)	n.a.	n.a

Family origin: Iraq (30%); Lebanon (25%); Balkans (17%); other Near and Middle East countries (Iran, Jordan, Syria) (11%); Somalia (9%); other countries (Afghanistan, Pakistan, Algeria, Egypt, Libya, Turkey, Italy, Slovakia, Chile, and South Africa) (8%)

The place of birth was known for all participants: see Table 1 for more information. None of the parents had been born in Sweden, therefore we considered it fair to describe the families as immigrant families. All children that had been born outside of Sweden (a small number) entered the country well before their second birthday.

### Environmental exposures

As expected, many apartments in the study area showed multiple structural defects with signs of moisture and mould growth very frequent (more than 50% of all apartments). The agreement between subjective and objective assessments was only fair to moderate. Parents were asked whether any persons living in the apartment were smoking inside. This was confirmed in 48 out of 130 households. At least 53 children (15% of all children in the study) were exposed to environmental tobacco smoke in their homes.

84 households (65%) were classified as crowded: more than one person per bedroom. See also Table 1. Crowded living conditions showed a significant association with both subjective and objective reports of dampness and mould.

48 out of 130 households (37%) reported current or previous presence of cockroaches.

### Skin prick tests

See Table 2 for detailed information. SPTs were performed in all seasons; positive results for seasonal plant allergens were not observed more frequently during the spring or summer months. Almost all - 115 out of 130 families (88%) - had a SPT performed for at least one family member. Overall, almost two thirds of all participants had SPTs performed (416 out of 650,). Eighty-seven SPTs (21% of all performed SPTs) were valid and positive for one or more antigens. Among all children between 0 and 13 years, 38 (24 in boys and 14 in girls) had positive SPTs. Skin prick testing in young children can be challenging and difficult to interpret not only for technical reasons, but also because of different immune characteristics of the skin itself at young age. Of the 359 children in the study, only 10% were under the age of 2 years (16 under 12 months old, and 19 between 12 and 24 months. Of these youngest children, only 6 underwent skin prick testing, with no positive response in any of them.

**Table 2:** Skin prick tests on study participants

	All Children (0-13 yrs)	male children (0-13 yrs)	female children (0-13 yrs)	older siblings	mothers	fathers
N	359	190	169	61	130	100
SPT done n (%)	232 (65%)	118 (62%)	114 (67%)	26 (65%)	106(74%)	52(48%)
SPT positive n (% of performed)	38 (16%)	24(20%)	14 (12%)	7(27%)	29(27%)	13(25%)
Monosensitized (% of pos.)/polysensitized	27/11	17 (71%) /7	10 (71%) /4	2 (29%) /5	16 (55%) /13	8 (62%) /5
Reaction to timothy grass n (% of positive tests)	12(32%)			24(49%)		
Reaction to birch n (% of positive tests)	8 (21%)			16 (33%)		
Reaction to HDM (D.far/pter) n (% of pos.)	13(34%)			11(22%)		
Reaction to animals (cat, dog, horse) n (%of pos.)	8 (21%)			7 (14%)		
Reaction to moulds n (% of positive tests)	7 (18%)			3 (6%)		
Reaction to cockroach	4 (11%)			4 (8%)		

Subjective reports of dampness or mould in the apartments were not related to the risk of the children living in the affected apartments to have a positive SPT. However, if the objective assessment of the apartment showed moisture and mould in the bathroom, the affected children had a higher likelihood of being sensitized than children who lived in apartments without these exposures (*p* = 0.017 and 0.022 respectively). Living in crowded quarters did not significantly influence a child’s risk for being sensitized in the SPT. Reported cockroach exposure bore no statistically significant relation to the inhabitants’ likelihood of having a positive SPT result overall. The participants who showed sensitization against cockroach allergen however were much more likely to live in an apartment with cockroach exposure (*p* = 0.006) than non-sensitized participants.

#### Children with positive SPTs

Well over half of the children with positive reactions were polysensitized. For further details see Table 2.

Children with positive SPTs were significantly older than the children who had no reactions (8.9 ± 2.9 vs. 6.8 ± 3.8 years (*p* = 0.002); this statistically significant difference was seen for both sexes. 4 of 55 children with a diagnosis of asthma from the PHC had positive reactions. Durations of total and exclusive breastfeeding were not different between the children with positive or negative SPTs. The number of older siblings did not affect the likelihood of a positive SPT. The average age of the mother at the time of birth of the child showed no difference between children with positive or negative SPTs.

#### Other participants with positive SPTs

Among parents and older siblings, IgE-mediated sensitizations were most frequently directed against plant antigens and house dust mites – see Table 2. Reactions to mould were much less frequent than in children.

The sensitizations seen in members of the same family did not show a discernible pattern (data not shown), i.e. there was no indication that sensitizations against particular antigens or even groups of antigens were preferentially passed along from parents to children.

There were 38 children whose father had a positive SPT, and 192 children whose mother had a positive SPT. 24% of the children with SPT-proven paternal atopy, and 12% of the children with SPT-proven maternal atopy had a positive SPT themselves. When comparing these children (with parents with positive SPTs) to those without parental atopy, only the presence of paternal atopy conferred a significantly increased risk of having a positive SPT to the children (*p* = 0.047 for paternal atopy vs. *P* = 0.687 for maternal atopy).

#### Selected antigens

Eleven participants showed a reaction against cockroach antigen. Cockroach is not part of the standard antigen panel used in regular primary care allergy testing in Sweden. Six of the eleven persons with a positive reaction to cockroach antigen were parents, and one was an older sibling, aged 20 years; all had been born abroad. Three of the four sensitized children had been born in Sweden and had not spent any relevant amount of time in the family’s country of origin. All homes with children who were sensitized against cockroach antigen reported having had cockroach infestation in the past, for which renovations had been undertaken.

#### Parental atopy

All families were asked whether the parents had asthma, allergic rhinitis, allergic eye symptoms, pollen allergy or atopic eczema. Twelve mothers and 21 fathers self-reported asthma. Allergic rhinitis was reported by 6 mothers and 1 father, allergic eye symptoms were reported by 4 mothers and one father each, pollen allergy was claimed by six mothers and nine fathers. All parents indicated that their symptoms had started several years after arrival in Sweden. They also reported that they had not received care in regard to these atopic symptoms from the primary healthcare system. Self-medication with OTC antihistamines was relatively common.

#### Questionnaire data on atopic diseases in children and correlation with other data

The parents were asked whether their children had ever been diagnosed with asthma by a physician: this was indicated in 83 children for asthma; of these, 65 were children with documented diagnoses from the healthcare records: these were asthma (46), acute bronchitis (7), infection-triggered asthma (7) and asthmatic bronchitis (5). For all 85 children with documented diagnoses from the healthcare records, these index diagnoses had been asthma (55), acute bronchitis (17), infection-triggered asthma (8) and asthmatic bronchitis (5). In other words, of the 55 children who had a diagnosis of asthma from the primary health care centre, this was only explicitly known to the parents in 46 cases (84%). It is not known how diagnoses were established and communicated to the parents and how well these were able to understand the information and integrate it into their health literacy. When considering parental reports of whether their child had regularly been using asthma-medication (inhalers), this was reported for 54 children. Parental reports of asthma diagnoses and medication use coincided for 51 children. However in 10 children the parents reported a diagnosis of asthma and no specific medication use. Medication use without a diagnosis of asthma was reported for three children.

## Discussion

One important finding is that this immigrant population was exposed to a multitude of potentially harmful factors connected to the built environment. Crowded households were the rule rather than the exception. Previous research has pointed out a strong inverse relationship between domestic crowding and atopic sensitization in children [29]. This was however not seen in our population. Pooled data from 5 cross-sectional asthma surveys has previously revealed that the effect of low socioeconomic status and pests in the household on asthma diagnosis differed for children born in- or outside the US [30], supporting the existence of either protective, or detrimental factors, or both, acting on these children.

A diagnosis of asthma can be notoriously difficult to establish and wheezing in younger children is more often associated with respiratory infections, while in older children, it is more often related to asthma. We did not attempt to establish verification of asthma diagnoses, but rather to capture a real-world picture of issues encountered in primary healthcare.

A positive skin-prick test (or for that matter a positive RAST) is no disease entity in itself; rather, it signifies an IgE-mediated sensitization against (environmental) allergens and as such can be an indicator of the presence of atopic disease states. Correlation with clinical manifestations is always required for assessment of significance. The increased risk for positive SPTs among children that was associated with positive paternal SPTs in our study is unlikely to be due to paternal selection bias, as the rate of positive tests among the tested mothers was comparable to that of the tested fathers: roughly 1 in 4 each. In the literature there has been considerable debate on whether maternal or paternal factors are more influential in the heredity of asthma and atopy, with stronger effects of maternal sensitization and/or history reported [31–35].

Sensitization to cockroach was seen in only a few individuals, and there is possibly an association with exposure in the home environment. While it does not seem justifiable to routinely test individuals with an immigrant background for this sensitization, clinicians should be aware of the possible role of cockroach antigen, especially when exposure is confirmed, and potentially in cases of difficult-to-treat-asthma in some individuals [36]. Studies in international adoptees in Sweden have shown that the age at immigration is of marked importance, with asthma incidence lower in case the arrival in Sweden occurred after the age of 2 years [37, 38]. Data from the U.S. showed that Mexican-American children who lived in the U.S. in their first year of life were more likely to have physician-diagnosed asthma than their peers who had lived in Mexico during the first year of life [39], and children born in the US had more wheezing, and a different profile of atopic sensitizations [40]. This was also seen in another analysis of the ISAAC data where a protective effect of having been born outside the country of residence also depended on having moved after age 2, and only when the move was into an affluent country [41]. Relating this to our results, we did not observe a protective effect of having been born abroad, presumably because all the children in our study came to Sweden very shortly after birth. Data from the UK indicates that the frequency of physician-diagnosed asthma may be lower in immigrant children, but their risk of hospital admission may be higher, probably influenced by differences in health-seeking behaviours or difficulties in accessing high-quality primary care services [42]. In a Swedish study from 1992 [43] the spectrum of allergic sensitization was found to be comparable between immigrants and Swedish controls, especially with longer time of residence. The prevalence as well as the spectrum of atopic sensitization to the new environmental allergens of the host country increases with time of residence, eventually approaching that of the autochthonous population [44]. Birch and timothy grass pollen sensitizations were seen frequently in both groups [44], but sensitizations against animal dander were more frequent among the Swedes. Research from Italy showed a high prevalence of atopic disorders in immigrants from extra-European countries, with a similar spectrum of sensitization to the Italian population, but possibly greater severity and among children more prevalent allergic rhinitis [45–48]. A further study in adoptive children examined soon after arrival in their new home country showed sensitizations (based on serum IgE analyses against food and inhalant allergens in 30 and 34%, respectively [49].Overall our results on sensitizations are in line with those reported in the literature.

The considerable discrepancies between children’s asthma diagnoses noted by the primary healthcare system, and parent’s knowledge of these diagnoses and administered treatments is a concern, and must raise questions about how diagnoses are generated and then disseminated, in order to ensure an appropriate degree of information among parents about their offspring’s medical conditions, which will affect their ability to care for their children. In view of the results presented here and in conjunction with research from other countries [41, 44, 48, 50] it would seem prudent to better prepare and equip primary health care services with the means to diagnose and treat atopic disorders among immigrants. Education of families about asthma and its best treatment is an empowering cornerstone of high-quality primary care provision for children in their social context. The importance of this approach is underscored by a careful study from Sweden, where neighbourhood deprivation level affected the risk of hospitalisation for childhood asthma independent of maternal socio-demographic characteristics [31].

One of the strengths of our study is the use of health communicators for home visits. These were not only familiar with the local conditions in the neighbourhood, but also fluent in the language spoken by the participants. The home visits were generally very well accepted by the participating families. Yao et al. [51] employed a very different methodology, in that they analysed data from a comprehensive health survey of the Canadian populace, exploring associations between immigrant status and atopic health information. Our own study was conducted at a more immediate and close-up level, also relying heavily on information verbally gathered from participants; we considered selection bias, but could not identify relevant differences between participating and non-participating families.

The immigrant population we studied is rather unique in a Swedish context, and not representative of the general Swedish population. We assume however, that there is a degree of generalizability to other immigrant populations in precarious housing conditions both in Sweden and in other affluent countries.

As in any study using self-reported sensitive information from marginalized populations, there are limitations with regard to the completeness and reliability; which would not be overcome by using official population registry data. Missing data was not imputed, complete cases only were analysed, and we cannot exclude having introduced a bias this way. Another limitation is that we lacked data on children’s daycare attendance, which has been investigated in relation to the development of atopic disorders [8, 9], presumably via increased numbers of respiratory infections in early childhood, which could steer the immune system away from an atopic path.

## Conclusions

The atopic burden in this selected immigrant population was high, and results indicate unmet medical needs. The pathways leading to the development and manifestation of atopic disorders are highly complex, and our results point to the presence of patterns where potentially harmful factors interact with potentially protective factors. Careful history-taking and attention to the social context remain very important tools for the practising allergologist and general practitioner.

Cross-sectional studies (even when they have some characteristics of a population-based study, like ours) have inherent limitations when trying to establish linkages between environmental exposures and health outcomes; such study designs are however valuable for exploratory analyses. As mentioned above, our aim was to establish a real-world picture of the burden carried by this population in order to better equip health care providers with information on what is necessary to improve care for such populations. Comprehensive asthma and allergy care for families will always require evaluation of multiple dimensions including the home environment. Home visits are a very valuable tool, and should be planned for by health care services attempting to treat people in their social context. This approach was also used in a population-based study in Colorado [52] in which adverse environmental housing conditions were identified in a significant proportion of recent Mexican imigrants’ homes, partially explaining asthma and atopic symptoms. In our study we could only demonstrate few statistically significant associations between objective exposure to “bad housing”, and the likelihood of atopic sensitization. Yet, in general terms, and following the precautionary principle, it is not acceptable for human beings to live in deficient housing. Even if biological pathways remain largely unexplained, and causal associations cannot be proven with statistical significance in individual cases, there is an imperative for action if clinicians and other health care providers are made aware of problems with the built environment. In fact they should actively seek such information. However, effective action is dependent not only on developing appropriate referral pathways in the healthcare system but also on communicating and cooperating with stakeholders across sectors in local and regional networks to effectively address such a systemic issue.
